# The Role of Social Media in Recruiting for Clinical Trials in Pregnancy

**DOI:** 10.1371/journal.pone.0092744

**Published:** 2014-03-26

**Authors:** Mahvash Shere, Xiu Yan Zhao, Gideon Koren

**Affiliations:** 1 Leslie Dan Faculty of Pharmacy, University of Toronto, Toronto, Ontario, Canada; 2 The Motherisk Program, Division of Clinical Pharmacology and Toxicology, Toronto, Ontario, Canada; 3 Child Health Evaluative Sciences, The Hospital for Sick Children, Toronto, Ontario, Canada; The National Institute for Health Innovation, New Zealand

## Abstract

**Background:**

Recruitment of women in the periconceptional period to clinical studies using traditional advertising through medical establishments is difficult and slow. Given the widespread use of the internet as a source for medical information and research, we analyze the impact of social media in the second phase of an ongoing randomized, open-label clinical trial among pregnant women. This study aims to assess the effectiveness of social media as a recruitment tool through the comparison of diverse recruitment techniques in two different phases of the trial.

**Methods:**

Recruitment in Phase 1 of the study consisted solely of traditional healthcare-based sources. This was compared to Phase 2 of the study where traditional recruitment was continued and expanded, while social media was used as a supplementary source. Yearly recruitment and recruitment rates in the two phases were compared using the Mann Whitney U test. The contributions of each recruitment source to overall recruitment were analyzed, and the impact of potential confounders on recruitment rate was evaluated using a multiple regression and Interrupted Time Series Analysis.

**Results:**

In the first phase of the study, with over 56 months of recruitment using traditional sources, 35 women were enrolled in the study, resulting in a mean rate of ±0.62 recruits/month. In the 6 months implementing recruitment through social media, 45 women were recruited, for a 12-fold higher rate of ±7.5 recruits/month. Attrition rates remained constant, suggesting that social media had a positive impact on recruitment. The Interrupted Time Series Analysis detected a significant difference in recruitment after the intervention of social media (p<0.0001) with an evident increase in the number of recruits observed after the use of social media.

**Conclusions:**

Clinicians and scientists recruiting for clinical studies should learn how to use online social media platforms to improve recruitment rates, thus increasing recruitment efficiency and cost-effectiveness.

## Introduction

Poor recruitment is a major obstacle to the successful and efficient completion of clinical trials. A recent survey of corresponding authors of randomized trials found that nearly 60% had either failed to meet their recruitment target or required an extended recruitment period [1]. Insufficient recruitment of study participants may result in losing the statistical power of a predictive conclusion, as well as prolonging the time and increasing the cost associated with the study.

The path to recruitment is usually the untold story in randomized clinical trials. While even failed results and conclusions of experiments are reported, inefficient recruitment methods often go unreported. Studies assessing effective recruitment strategies are far too scarce. The few systematic reviews that have addressed this issue stress the lack of generalizability of recruitment methods given the degree of subjectivity with respect to a particular study design, intervention type, and the nature of participation required by volunteers [1]–[4].

The issue of poor recruitment becomes even more exaggerated when the target of a study is a special population such as women in the periconceptional period or during pregnancy. Risk perception with a clinical intervention during this period is often skewed from ‘actual risk’ to ‘imagined risk’ given this state of vulnerability and fears of coercion [5]. As a result, there is a great need for the assessment of recruitment strategies in special populations, such as women in the periconceptional period, which are not only efficient but also cost-effective.

With the advent of the internet and medical information being available on the internet in recent years, volunteers participating in clinical trials have moved away from being “patients” to “informed health-care consumers” [6]. Many people thoroughly search their symptoms on the internet before they decide to visit a physician who assigns them a diagnosis. About one-third of American adults access social media for health matters [7]. A survey conducted by the Opinion Research Corporation demonstrated that 59% of adults in the USA use the internet to seek health information [8], making it the most popular option over seeking similar information from a healthcare provider.

**Figure 1:**
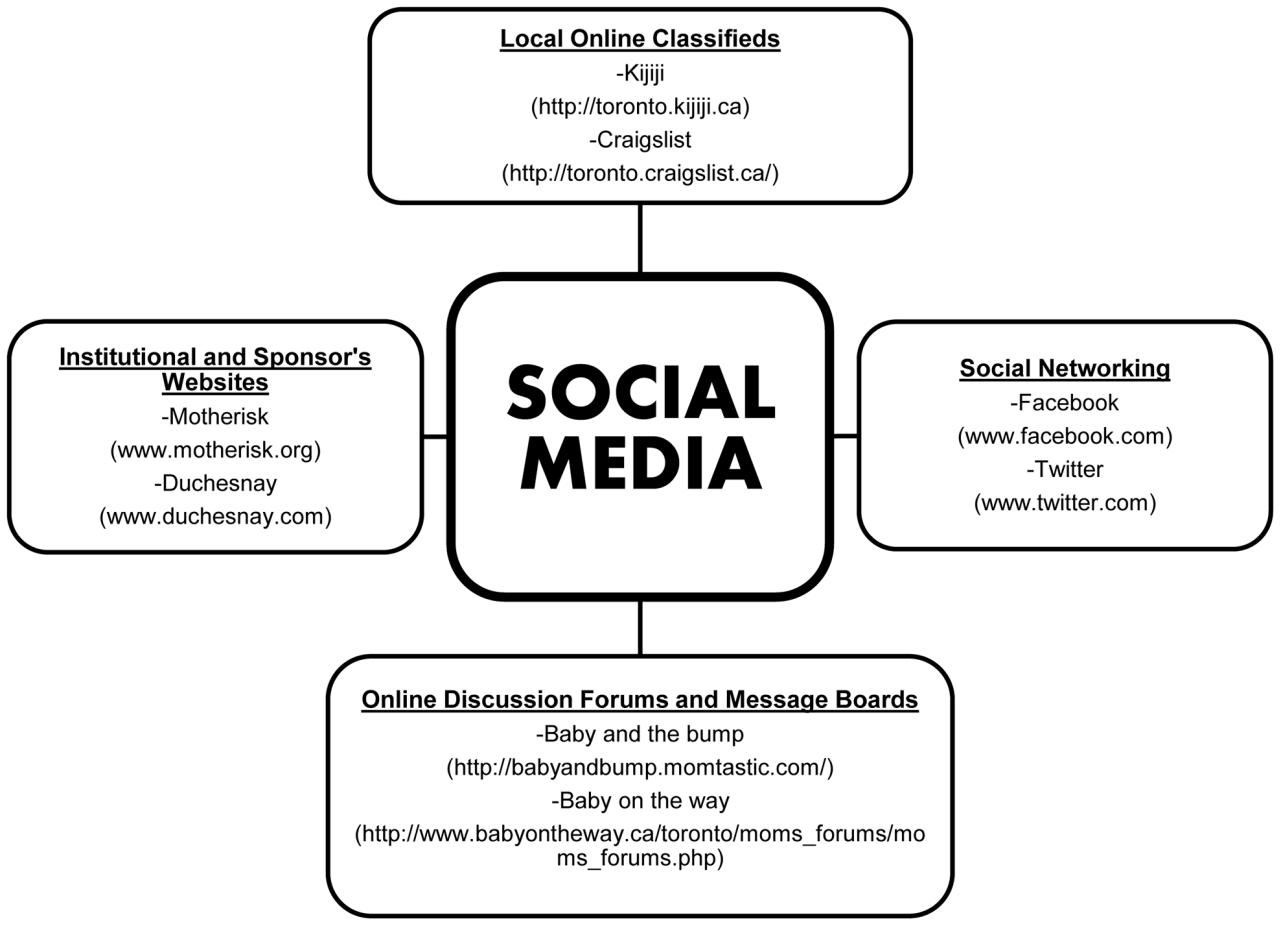
What constitutes Social Media? The various online social media and networking platforms used for the recruitment of pregnant and planning women in the study.

The accessibility of medical information on the internet has not only made modern-day patients more aware, but also more involved in their personal healthcare. Thus, using social media to expose clinical trials to a larger subject population seems like an obvious next step in trying to optimize recruitment strategies. **Social media** is typically ***an online platform that can enable dialogue among individuals and online communities, serving as a site for information dissemination and discussion.*** After several years of slow and frustrating recruitment for a randomized, open-label trial on folic acid in pregnancy, we decided to examine the effectiveness of recruitment through social media. We use a broad definition of social media which encompasses social networking sites such as Facebook and Twitter, along with local online city classifieds like Kijiji and Craigslist, as well as online discussion forums and message boards on specific websites (Figure 1). Though skepticism and issues with credibility of information on social media prevail on the minds of both participants and healthcare professionals, we hypothesized that social media may be a valuable tool for recruitment if used in an organized and targeted fashion. The objective of the present report was to compare recruitment success and efficiency between traditional healthcare-based methods of recruitment vs. social media.

## Methods

### Study

**Figure 2:**
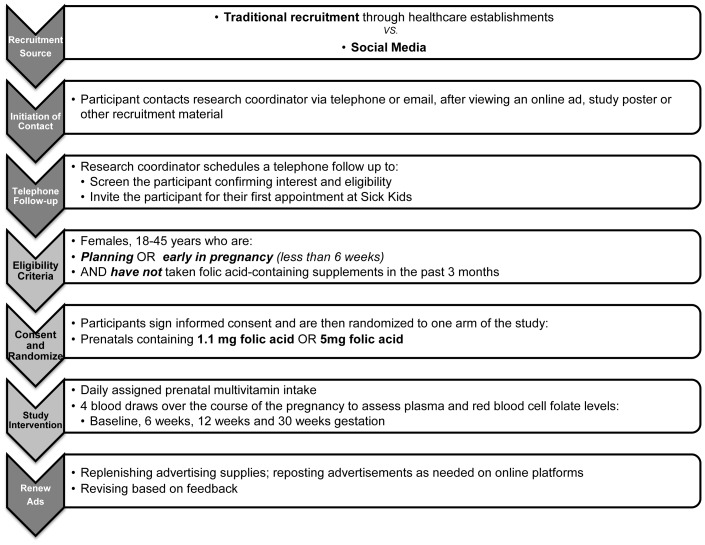
Study design. An outline of the study design.

This study is based on secondary, post-hoc analysis emerging from a randomized clinical trial with the objective of comparing the steady-state pharmacokinetics of regular (1.1 mg) and high (5 mg) folic acid tablets over 30 weeks of pregnancy. The study aimed at recruiting women between the ages of 18–45 years, who were not taking folic acid-containing multivitamins 3 months prior to enrollment, and were either early in pregnancy or trying to conceive. Women who had a previous history or a previous affected pregnancy with neural tube defects were excluded from the study (Figure 2). Participants who completed the study were granted monetary compensation for their participation. This was pro-rated based on the degree of participation and remained the same over the course of the study.

### Recruitment

Originally, recruitment materials such as posters, ads and study information brochures were approved by the Hospital for Sick Children Research Ethics Board for the study in 2007.

In the first phase of the study (from April 2007–November 2011), the primary mode of recruitment focused on recruiting participants amongst healthcare establishments. This was achieved through a variety of different methods. We targeted women who called the Motherisk program. The Motherisk program is a telephone counselling program that provides evidence-based information to healthcare professionals and women about the safety and teratogenic risks associated with drugs, chemicals, radiation, infections and other exposures in the periconceptional period. Due to the large volume of callers every day, study details were presented to women who fit the inclusion criteria by Motherisk counsellors and they were asked for telephone consent and follow-up by the study coordinator if they expressed interest in participating. Motherisk counsellors were trained to briefly explain the main objective, intervention, time course and target population of the clinical trial to callers who expressed interest. Interested callers were then followed up by the study coordinator. Voicemails were left after initial consent if interested callers could not be reached, and were called back up to a maximum of three times. Concurrently, we used notice board postings at Sick Kids and Women’s College hospitals in areas where a general high traffic of families was expected. The notice board postings at both these hospitals were re-posted monthly or as needed after approval from Public Relations department. Furthermore, study materials were presented in the form of brochures and postings before and after clinic appointments, to eligible women at the above hospitals as well as fertility clinics associated with the study. This was achieved through multiple ways. Study brochures were available within waiting rooms and study postings were also posted on bulletin boards within the clinics. Secondary staff, who usually included research coordinators at other clinics or hospitals, closely communicated with the folic acid study coordinator to identify eligible patients arriving at the clinic for the upcoming week. Patients were screened on the basis of the inclusion criteria, with the major determiners being women who were “early in pregnancy” or “trying to conceive within 3 months” and “were not currently on folate-containing multivitamins”. Eligible patients were flagged based on the review of patient charts each week and upon their clinic visit, study details were presented to them through two possible ways: either through physicians at the clinic during their consultation, or, if this was not possible due to time-constraints or other commitments of an appointment, physicians often directed flagged patients to secondary staff at each clinic, who were trained to briefly outline study information and were able to spend more time with each patient reviewing the objective and demands of the study. In both these cases, upon interest, a women was handed a study brochure outlining study information and the contact information of the study coordinator. Interested women were asked to contact the study coordinator directly via telephone or email, as listed on the brochure. Monthly meetings were held at all clinics between the study coordinator and the secondary study staff to go over recruitment progress and replenish advertisement supplies. All study postings were also revisited monthly or as needed for renewal.

After the study had been going on for 4 years, recruitment strategies were re-evaluated in the second phase of the study (December 2011–May 2012). We continued to actively engage in recruitment approaches based amongst healthcare establishments. These included pregnancy and prenatal community programs and health centers, family doctor’s offices, new immigrants and women’s centers, university health clinics, and midwifery clinics, as well as all of the previously used recruitment sources. As before, women interested in participating could contact the study coordinator via telephone or email, as listed on the advertisement.

However, in parallel to this approach, advertising was expanded to the realm of social media. Study details were posted on the drug sponsor’s website, Duchesnay (www.duchesnay.com) and the Motherisk website (www.motherisk.org), both of which are largely accessed by women in the periconceptional period. Advertisements were also posted regularly on local online classifieds such as Craigslist (http://toronto.craigslist.ca/) and Kijiji (http://toronto.kijiji.ca) in the “community, volunteers” and “baby items/baby+kids” categories to target women planning families. Ads on these platforms were renewed every 2–3 days to keep them well updated on the front page of each section. A similar approach was used with study postings on pregnancy discussion forums and message boards, such as *Baby and the bump* (http://babyandbump.momtastic.com/) and *Baby on the way* (http://www.babyontheway.ca/toronto). Study ads were created as new threads or announcements, and were renewed every month, or as needed. Further, postings detailing study information were also occasionally posted on social media networks such as Facebook (www.facebook.com) and Twitter (www.twitter.com). Posts on all of above online media were limited to placement of study ads, as approved by the Ethics Review Board at the Hospital of Sick Children. Interested participants were asked to contact the study coordinator if they had any questions or concerns, which were addressed via telephone follow-up, as per the study protocol.

Upon approaching the study coordinator with interest in the study, the participants were contacted by a healthcare professional member of the study to discuss study details and confirm consent to participate. Interested participants were then invited to the Hospital for Sick Children for their first appointment to go over formalized informed consent and proceed further with the study (Figure 2).

### Statistical Analysis

Recruitment from the two phases of the study was divided such that Phase 1 included “traditional” recruitment from healthcare establishments, whereas Phase 2 included “traditional” recruitment supplemented with “social media”. All data were tested for normal distribution. We compared monthly recruitment between the two phases using the Mann Whitney U test, since the data were not normally distributed. Furthermore, the recruitment rate per year was calculated using the ratio of women recruited over the period of time that recruitment was conducted. Characteristics of the women in the two groups (“traditional” vs. “social media”) were compared by the chi-square test or Student’s t-test, wherever applicable. All of the above analysis were conducted using GraphPad Prism (version 5; GraphPad Software, San Diego, CA).

A multiple regression was conducted using IBM SPSS (version 18; PASW Statistics, Armonk, NY) to analyze the impact of predictors such as recruitment source, age, previous fertility problems and a previous history of chronic illnesses on recruitment rate. Given our sample size of 80 participants, we identified the four most important predictors affecting recruitment rate and used these as covariates within the multiple regression.

As part of the multiple regression, the Durbin-Watson statistic was evaluated, to assess the independence of residuals.

Interrupted Time Series Analysis was conducted using SAS (version 9.3; SAS Institute, Cary, NC) to detect a difference in recruitment after the introduction of social media. Log transformation of the raw values was used to transfer the data counts close to normal distribution. Visual inspection of the series plots, autocorrelation functions (ACF), partial autocorrelation function (PACF), inverse autocorrelation function (IACF) plots, and Dickey-Fuller unit root tests were used to check the assumption of stationarity. Non-significant results showed stationarity of the series; therefore, no differencing was used.

The shifted mean was modelled by creating a shift indicator with the value of “0” or “1” before and after the intervention of social media. Transform function was used to realize it within the model building process.

The error structure was then fitted with the autoregressive integrated moving average (ARIMA) model. The order of *p, q*, and *d* in ARIMA model was determined by carefully examination of ACF, PACF, and IACF plots. Maximum likelihood method was used for estimation of parameters.

Chi-Square test on the residuals was used to assess if series was left with white noise. Non-significant Chi-Square statistics indicated that our model fitted well. Residual QQ plots were used to test the departure from normality assumption.

A set of candidate models were arrived at, and final model was selected by the lowest akaike information criterion (AIC), significant parameter estimates (indicator and AR/MA lags), Chi-square test on the residuals, residuals diagnosis plots, and forecast plots. Fitted plots were produced in both log and original scale.

## Results

**Figure 3:**
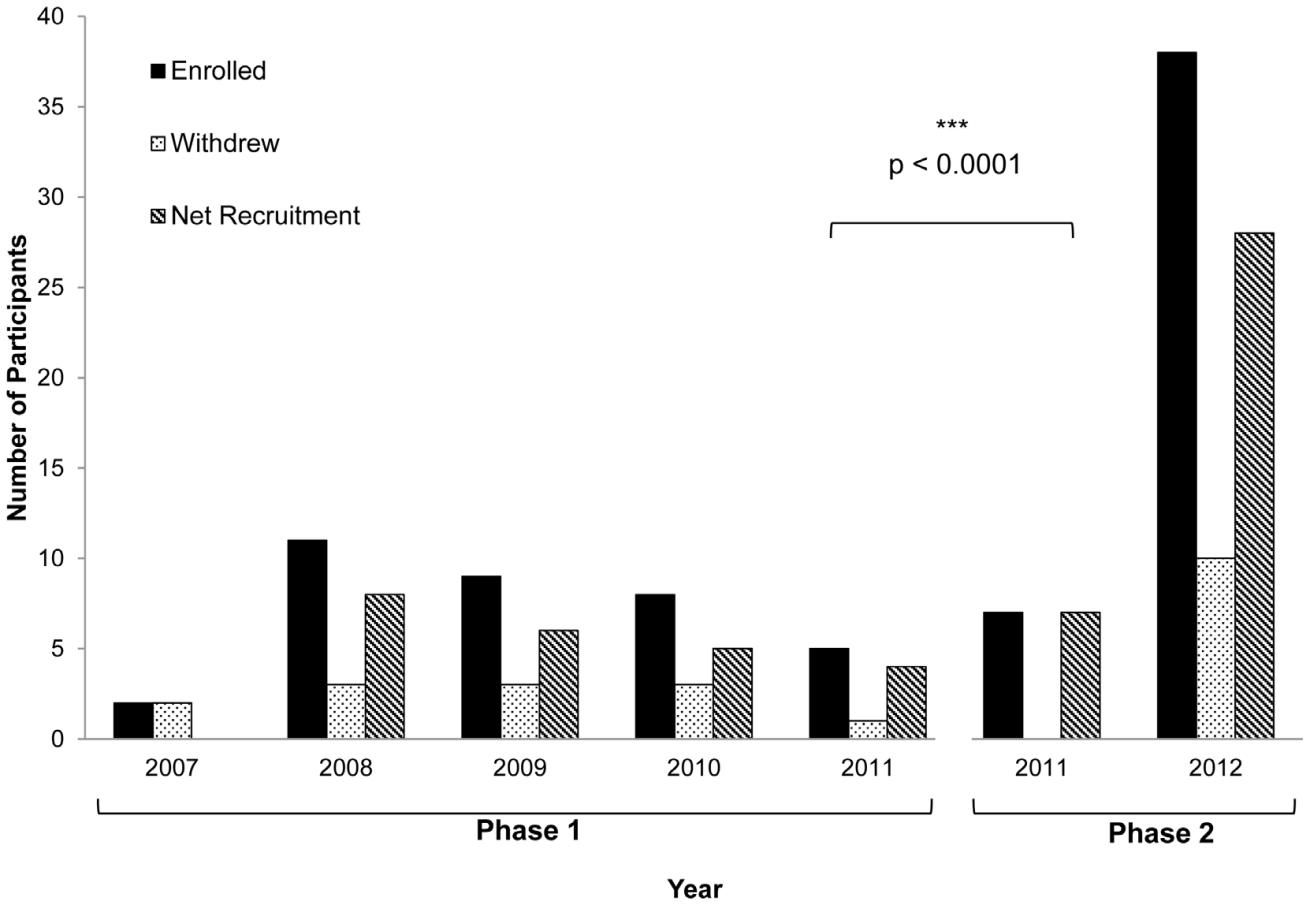
Success of recruitment strategies over time. Recruitment through traditional healthcare-based advertising constituted the first phase of recruitment from April 2007–November 2011. Starting December 2011–May 2012, new social media based recruitment strategies were applied along with continued use of traditional healthcare-based recruitment. Yearly recruitment in the two phases was compared using the Mann Whitney U test.

**Figure 4:**
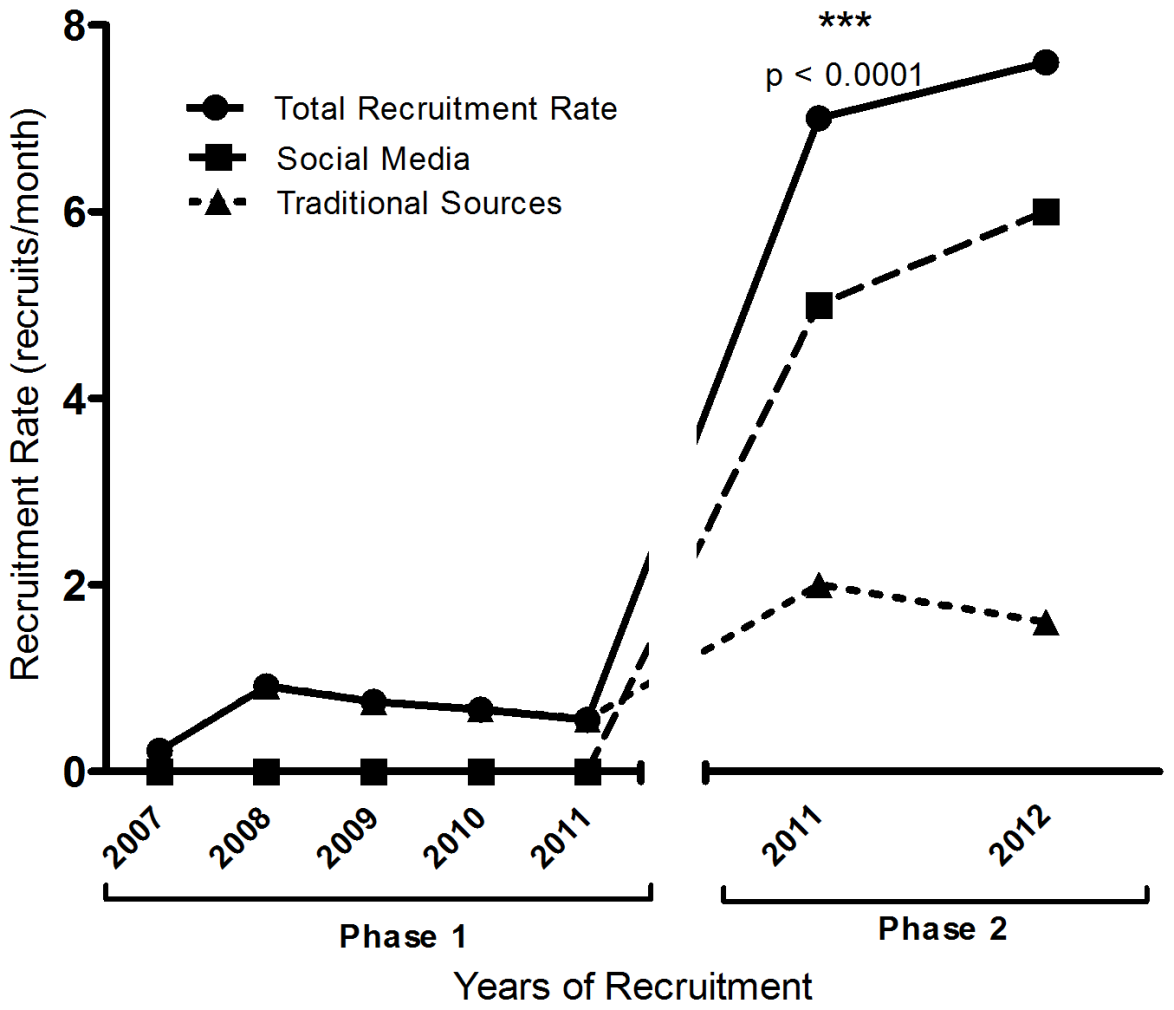
Recruitment rate over time. Recruitment through traditional healthcare-based advertising constituted the first phase of recruitment from April 2007–November 2011. Starting December 2011–May 2012, new social media based recruitment strategies were applied along with continued use of traditional healthcare-based recruitment. Recruitment rates in the two phases were compared using the Mann Whitney U test.

The study started recruitment in April 2007. Between 2007–2011, with over 56 months of recruitment in Phase 1 of the study using traditional sources, 35 women were enrolled in the study, resulting in a mean rate of ±0.62 recruits per month. In Phase 2 of the study, ongoing recruitment from traditional sources was supplemented with active recruitment from social media-based sources. During these 6 months implementing recruitment through social media (December 2011–May 2012), 45 women were recruited, for a 12-fold higher rate of ±7.5 recruits per month (p<0.0001) (Figure 3 and 4). Despite the fact that traditional healthcare-based recruitment outlets were expanded, social media generated about 78% of the recruitment during this phase. Amongst these sources, local online classifieds such as Kijiji and Craigslist had the greatest contribution of 58% of total recruitment (Table 1).

**Table 1:** Breakdown of Recruitment.

	Advertisementplatform	Recruited	Approached (estimatedaverages where applicable)	Percent of totalrecruitment (%)	Recruitment rate(recruit per month)
**Phase 1** **(n = 35, t = 56 mos)**	Motherisk calls	26	41	74.3	0.46
*Traditional Healthcare* *Establishments*	Fertility clinics	4	12	11.4	0.07
	Hospital postings	5	High-traffic areas	14.2	0.09
**Phase 2** **(n = 45, t = 6 mos)**	Kijiji	11	25–38 hits per post	24.4	1.83
*Social Media*	Craigslist	15	n/a	33.3	2.50
	Motherisk website	6	n/a	13.3	1.00
	Facebook	2	n/a	4.4	0.33
	Twitter	0	n/a	0	0
	Online discussionboards	1	30–40 views per post	2.2	0.17
**Phase 2** **(n = 45, t = 6 mos)**	Communityhealthcare sites	3	High-traffic areas	6.7	0.50
*Traditional Healthcare* *Establishments*	Motherisk calls	4	13	8.9	0.66
	Fertility clinics	2	8	4.4	0.33
	Hospital postings	1	High-traffic areas	2.2	0.17

The ratio of withdrawals adjusted for total recruitment maintained a constant trend in both phases, as it was 0.34 during the first 56 months, and 0.22 during the 6 months of the second phase (Figure 3 and 4).

The women recruited by the two methods were not different significantly by any of the assessed variables including body weight, age, gravidity, race distribution, marital status, level of education and employment (Table 2).

**Table 2:** Characteristics of women recruited by traditional methods vs. social media.

	Traditional Healthcare Establishments (n = 35)	Social Media (n = 45)	p
**Time spent using method** (mos)	56 mos	6 mos	**–**
**Age** (yr)	31.3±3.85	31.7±4.83	**0.73**
**Weight** (kg)	68.9±17.7	63.9±11.6	**0.23**
**Gravidity**			**0.38**
- 0	8	10	
- 1	9	15	
- 2	5	4	
- ≥3	1	6	
**Ethnicity**			**0.81**
- Caucasian	13	17	
- Black	1	5	
- Asian	3	3	
- Hispanic	2	5	
- South Asian	3	4	
- Other	1	1	
**Marital Status**			**0.16**
- Single	0	3	
- In a relationship	1	5	
- Engaged	1	0	
- Married	21	27	
**Highest Level of Education**			**0.28**
- High school	2	5	
- College	2	9	
- University	11	14	
- Post-graduate	8	7	
**Employment**			**0.29**
- Full time	16	20	
- Part time	3	7	
- Student	0	4	
- Unemployed/homemaker	4	4	

The multiple linear regression confirmed social media as a significant predictor of recruitment rate (p<0.05) with a Pearson correlation of 0.81 and an R^2^ value of 0.656. The other predictors including age, fertility issues or a history of chronic illnesses did not statistically significantly predict recruitment rate. Regression coefficients and their standard errors are summarized in (Table 3).

**Table 3:** Summary of Multiple Regression Analysis.

Variable	B	SE_B_	β
Intercept	−98.065	25.379	
Recruitment source [0 = pre-social media, 1 = social media]	9.444	1.504	1.761* (p = 0.024)
Age	3.189	0.824	1.595
Fertility issues	−21.719	7.216	−0.767
Chronic illness	−10.093	3.688	−0.714

As part of the multiple regression, the Durbin-Watson statistic was evaluated, to assess the independence of residuals. We obtained a Durbin-Watson statistic of 2.56, indicating that the possibility of autocorrelation could not be ruled out. For this reason, time-series analysis was conducted.

**Figure 5:**
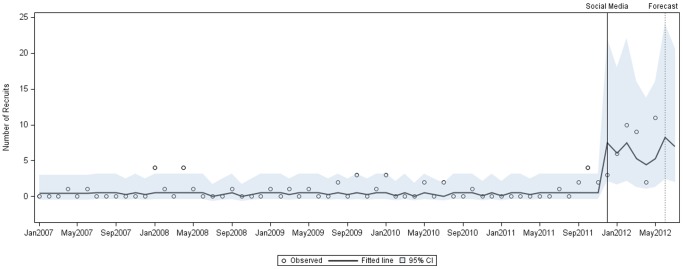
Interrupted Time Series: recruitment after the intervention of social media. Monthly recruitment *before* and *after* the intervention of social media, as observed and fitted with a logarithmic time-series model, within a 95% confidence interval. A forecast plot is also applied to predict the upward trend in recruitment observed after the intervention with social media.

After log transformation of the interrupted time series model, stationarity was established, and a shifted mean was determined for both phases of the study. No determined trend or seasonality was found, which may have been due to limited time points. Prior to the intervention of social media, the mean was 0.41, whereas after the intervention with social media, this mean shifted statistically significantly to 6.69 (p<0.0001), as obtained through Maximum Likelihood Estimation. The ARIMA model used had *d = 0, p = 1, q = 1*, and the chi-square test on residuals demonstrated that the residuals were merely random errors (p>0.05) and thus, the model was a good fit. Thus, the predicted model closely fit the observed data (as both are generally within the 95% confidence interval), with an evident increase in the number of recruits observed after the use of social media (Figure 5).

The final model used is as below:
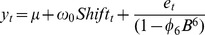
where *p* = 6, *q* = 0, *d* = 0 and *φ* is the autoregressive operator.

## Discussion

Given the limited research on effective recruitment strategies in clinical trials concerning special populations, especially women in the periconceptional period and pregnancy [2], [5], [10], this paper investigated the introduction of online social media as a targeted recruitment strategy to supplement traditional methods of recruitment. This study is the first to assess the efficacy of social media as the primary recruitment tool and its overall success compared to traditional health-care based recruitment amongst women in the periconceptional period.

In our study, net recruitment through health professionals, clinics, and health centers associated with the Motherisk program over the course of 5 years was 0.62 recruits per month. Although recruitment rate from traditional healthcare-based sources was doubled by adding new sources in Phase 2 of the study, this increase did not explain the significant increase in recruitment. Instead, the introduction of social media was strongly associated with a twelve-fold increase in recruitment rate in Phase 2. Analysis of socio-demographic characteristics between the two groups showed no differences, indicating a homogenous study population, thus alleviating concerns about selection biases based on different recruitment sources. Interrupted time-series analysis strongly endorsed the use of social media as the cause of this increase in recruitment, with a statistically significant increase in mean recruitment (p<0.0001) after the introduction of social media.

While recruitment rate is a key variable in determining how many participants will enroll in a study, assessing attrition rate [3]–[5] is also critical to ensure that maximum participants who enroll are being retained in the study, and there are a sufficient number of participants to eventually meet the sample size and power requirements for the study. We observed that the rate of withdrawals, adjusted for the total rate of recruitment, was constant over time.

While advertising on mass media platforms through radio and television announcements, as well as newspaper and magazine advertisements have been a recruitment approach used by many clinical trials in the past, these are generally passive recruitment strategies [1], [10] that often involve high costs which may not translate to a similar degree of returns.

The difference between these conventional forms of mass media and online social media seems like a subtle one at first, yet has immense implications. While recruits from other forms of mass media represent people who may have *landed upon* the research opportunity *by chance*, and then generated interest in it, recruits from social media are primarily people who have been *actively seeking* information about a related topic, whose pursuit leads them to a particular ad about a research opportunity. This is reinforced by public surveys that indicate that the internet is the first point of reference on the path to seeking health and wellness information for many people [6]. Hence, though the use of social media has traditionally been classified as a low-cost “passive” recruitment tool [9], we believe that the use of social media as recruitment tool should be redefined to “actively” target a specific population to yield highly efficient and cost-effective results.

In our experience, the key challenge faced in the first phase of recruitment was capturing women who were planning or early a pregnancy, yet *had not* already begun prenatal supplementation. Women being targeted at healthcare establishments seem to be self-motivated women who are already aware of all the right things to do before pregnancy, and thus many potential candidates did not fit our inclusion criteria if they had already begun taking folic acid.

In contrast, since many women use the internet extensively as a health-seeking source to research information in pregnancy before they are trying to conceive or during pregnancy, it is likely that they may have landed upon our study ad during their search, and we were likely able to capture eligible women on social media platforms *one step earlier* than we would have found them through traditional sources.

In our study, the social media outlets were chosen based on their wide reach and capacity to target a large volume of people every day. While general platforms such as Facebook, Twitter, Kijiji and Craigslist were chosen for their broad scope, advertising on these portals was streamlined to sections where a high traffic of women or families would be expected. This included the “volunteers” or “baby items” sections on online classifieds, and study postings through pages focusing on women’s health and pregnancy on Facebook and Twitter. Institutional and sponsor’s websites, as well as pregnancy forums and message boards, were similarly chosen because of the high traffic of women in the periconceptional period expected on these platforms.

Because of technological improvements and our consequent increased dependence on social media over time, recruitment for the study in 2007 vs. 2012 cannot be compared solely *through time*. However, as demonstrated by recruitment rates in Phase 2, even when recruitment through traditional sources was expanded and actively engaged, their contribution to recruitment was modest, while social media contributed significantly to the sharp increase in recruitment rates, thus *increasing the overall efficiency of recruitment*. It is also likely that awareness through social media contributed to general interest in the study, including word-of-mouth referrals as seen in other studies [9], [10], and may have influenced some of the recruitment from traditional healthcare-based sources.

Aspects of our study that may have contributed positively to recruitment, and are consistent with the literature on effective recruitment strategies [1] include: the inclusion of monetary incentive upon completion, a relatively low-risk intervention, as well as a potential previous knowledge or recommendation of the study drug. However, specific aspects that strongly influenced the positive impact that social media had on recruitment include the fact that our study was a health-seeking study targeting a population–likely young to middle-aged women–who are active users of social media platforms and are often using the internet as the first point in *seeking* this information. The marked impact of social media may not have been as significant if we were targeting an elderly population or patients suffering from certain chronic disease states. Thus, factors such as age, generational demographics and scope of internet access will inevitably define the reach, relevance and efficiency of social media as a recruitment source.

One of the most common apprehensions against using social media may include suspected bias in the population recruited from these platforms. As monetary incentive was advertised within the posting, there may be concerns that the population recruited may be more interested in money-making endeavours and may not be fully committed to the study, since platforms such as Kijiji and Craigslist (which contributed about 58% of recruits in Phase 2) are often used to find economical deals. However, comparisons of socio-demographic characteristics as well as a previous history of medical conditions and fertility issues between recruits from traditional healthcare-based vs. social media-based sources revealed no differences in our study. Due to our limitations of a small sample size, further research is necessary to investigate potential bias in the population recruited from social media-based platforms. Nonetheless, our results suggested that even though compensation may act as an initial attractor to invoke interest in pursuing a study, since participants were only given compensation upon completing the study, their continued participation indicated commitment to research for benefits beyond short-term monetary gain.

While privacy issues and legal concerns were not a challenge that we personally faced, they pose a great obstacle preventing most researchers from adopting social media-based recruitment approaches [6], [9]. Our approach was hence to use social media solely as a recruitment platform. The study material that was posted on social media sites was limited to advertising, and was identical to material that was disseminated through posters, brochures or postings using traditional healthcare-based recruitment sources. User comments and questions were not engaged with on public fora, but were instead redirected by asking them to contact the study coordinator via telephone or email, to maintain confidential interaction as per the study protocol.

In general, some of the limitations of our study included its small sample size and the small time course over which social media was applied. These were limited within our study because of the small sample size requirements of the clinic trial this is associated with. Yet this sample size was sufficient to demonstrate significant changes due to a large effect size. Further larger-scale research is warranted to explore the applied use of social media as part of larger clinical trials. Also, since the analysis on recruitment within this study was composed of secondary post-hoc analysis, further research is necessary to understand the broader applications of social-media based recruitment in clinical trials.

A white paper by Oglivy Washington and The Center for Social Impact Communication at Georgetown University outlines tenets on the use of social media for public health marketing [7]. Based on our experience in the current study, along with some suggestions modified from the paper above, we propose the following guidelines for using online social media as a primary cost-effective and efficient recruitment tool in clinical trials:

### 1) Understanding the Target Population

The primary step in the process of transforming the use of social media as a recruitment tool from “passive” to “active” is to thoroughly research the target population. Participants should not be treated merely as ‘the general population’. It is crucial to identify the defining characteristics and social networks of the population being targeted if it represents a special population (i.e. pregnant women vs. women battling cancer vs. elderly). This will not only help in creating population-specific platforms and goals, but also present early insight into the potential efficacy of recruitment through social media as an approach based on the internet use and medical-information-intensive use of a particular demographic. Even if the study merely aims to recruit “healthy volunteers” from the “general population”, the overall population should be segmented to different demographics, and each should be targeted individually for optimal results. In the age of internet access and the reliance of the population on the internet for medical information, it is crucial that we use online tools of social media to personally target and empower the participant, and move away from mere business and marketing approaches to recruitment where ads are created as ‘one-size-fits-all’ aimed to capture a random portion of the ‘general population’.

To truly recruit individuals who are interested in research for its own sake and the potential benefits it represents to them as well as the society at large, it is critical to approach participants with intentions of honest medical dialogue, equipped to address more complex questions given the plethora of information they are exposed to the internet.

Online social media, if used correctly, epitomizes community and conversation, and is a definite movement away from fears of coercion that may arise with the use of high incentives, direct interaction with the primary caregiver, as well as some other means of recruitment.

### 2) Using a Combination of Passive, Broad-spectrum, as Well as Targeted Active Recruitment Techniques

Any holistic recruitment strategy should incorporate a combination of active and passive recruitment techniques to maximize chances of success. Traditionally, active techniques have yielded greater participants, yet are also associated with a greater cost and time involvement [9], [10]. Passive techniques may be less efficient yet hold the potential for exposure to a larger population.

Social media incorporates both these characteristics when used in an applied fashion, and thus, may greatly enhance recruitment if used to supplement other recruitment approaches. Posts on social media platforms, depending on how intensively they are updated and the type of platform being used, hold hybrid characteristics of active and passive recruitment methods. In a space where one is targeting a specific demographic, they represent the active attempt to engage that specific subpopulation. However, on larger social networking platforms, they hold great power of dissemination given the large degree of exposure.

### 3) Monitoring Response Rates and Revising Methods based on Feedback

The organic and *live* nature of social media necessitates continual response-monitoring and the revision of strategies based on response. Since any updates on postings or edits literally appear real-time, it is important to evaluate the impact a particular recruitment strategy has on achieving target goals. Oftentimes, participants contact the study coordinator stating their confusion or reluctance with an aspect of the study based on the ad. All feedback should be recorded and appropriately incorporated to keep recruitment postings clear and up-to-date.

It is critical to keep regularly updating ads, ensuring that they are always around the top and have the chance to be most frequently read. If there is an obvious lag in response, potential limitations of the approach should be assessed and revised.

### 4) Addressing Apprehensions, Maintaining Transparency, and Transitioning the Participant Encounter to a Regulated Environment

With the breadth of medical information being available on the internet, there is also a breadth of *incorrect* information. Accordingly, some people may be suspicious of the credibility of a clinical trial attempting to recruit through online platforms that aren’t necessarily regulated by healthcare centers. To address this apprehension, it is crucial to state affiliations to the research institution [6], including contact information, so that contact is not only easy to establish but also secure for the participant.

While social media may serve as a prolific window to recruiting a participant, explanation of the study protocol and the process of informed consent should be executed at the research institution in a secure and regulated setting as per study protocol.

If the study involves online questionnaires or online completion of forms, secure databases should be used to maintain patient confidentiality and ensure security of patient information.

In conclusion, given that the recruitment through traditional medical establishments was intensively employed in both phases, and that the surge in recruitment rate was attributed mainly to social media-based recruitment methods, we can conclude that supplementing with social media-based recruitment strategies increase the efficiency of the recruitment process. Despite potential apprehensions, social media holds great promise as a recruitment tool if applied in a targeted and regulated fashion. Though the magnitude of its impact on recruitment may vary based on the sample size required, the study design, and the nature of its intervention, social media can be effectively incorporated in the recruitment efforts of any study to enhance current recruitment methods.
